# Selected Papers from the Ninth International Conference on Computational Intelligence and Security

**DOI:** 10.1155/2013/467321

**Published:** 2013-12-17

**Authors:** Yiu-ming Cheung, Yuping Wang, Hailin Liu, Xiaodong Li

**Affiliations:** ^1^Department of Computer Science, Hong Kong Baptist University, Hong Kong; ^2^School of Computer Science and Technology, Xidian University, Xi'an 710071, China; ^3^School of Applied Mathematics, Guangdong University of Technology, Guangzhou 510520, China; ^4^School of Computer Science and Information Technology, RMIT University, Melbourne, VIC, Australia

The 2012 International Conference on Computational Intelligence and Security (CIS) is the ninth one focusing on all areas of two crucial fields in information processing: computational intelligence (CI) and information security (IS). In particular, the CIS Conference provides a platform to explore the potential applications of CI models, algorithms, and technologies to IS.

Among all accepted papers in CIS Conference 2012, five papers were further screened and extended to be included in this special issue. They are the following:

(i) “Attribute index and uniform design based multiobjective association rule mining with evolutionary algorithm,”
(ii) “Reliable execution based on CPN and skyline pptimization for web service composition,”
(iii) “Robust adaptive control for a class of uncertain nonlinear systems with time-varying delay,”
(iv) “Bounds of the spectral radius and the Nordhaus-Gaddum type of the graphs,”
(v) “The effects of different representations on static structure analysis of computer malware signatures.”

The first paper formulates the association rule mining as a multiobjective problem, through which the algorithm of attribute index and uniform design-based multiobjective association rule mining with evolutionary algorithm is presented without the user-specified minimum support and minimum confidence anymore. Experiments on several databases have demonstrated that the proposed algorithm has excellent performance and that it can significantly reduce the number of comparisons and time consumption. The second paper is to employ the transactional properties and nonfunctional quality-of-service (QoS) properties for selecting the web services. Furthermore, the third paper presents an adaptive neural control design for a class of perturbed nonlinear MIMO time-varying delay systems in a block-triangular form. The proposed control guarantees that all closed-loop signals remain bounded, while the output tracking error dynamics converge to a neighborhood of the desired trajectories. The simulation results have demonstrated the effectiveness of the proposed control scheme. The fourth paper is to study the upper bounds for the spectral radius in quantum chemistry. As a result, an upper bound of the Nordhaus-Gaddum type is obtained for the sum of Laplacian spectral radius of a connected graph and its complement. Lastly, the fifth paper is to evaluate a static structure approach to malware modeling using the growing malware signature databases. It has been shown that it is possible to apply standard sequence alignment techniques in bioinformatics to improve accuracy of distinguishing between worm and virus signatures if malware signatures are represented as artificial protein sequences. Moreover, aligned signature sequences can be mined through traditional data mining techniques to extract metasignatures that help distinguish between viral and worm signatures.

To sum up, the previously mentioned papers will provide readers and researchers with some useful ideas in the fields of computational intelligence and security.

*Yiu-ming Cheung* *Yiu-ming Cheung* *Yuping Wang* *Yuping Wang* *Hailin Liu* *Hailin Liu* *Xiaodong Li* *Xiaodong Li*

